# Health-related quality of life of adolescents with sickle cell disease in sub-Saharan Africa: a cross-sectional study

**DOI:** 10.1186/s12878-019-0141-8

**Published:** 2019-05-14

**Authors:** David Muthahi Kambasu, Joseph Rujumba, Hervé Monka Lekuya, Deogratias Munube, Ezekiel Mupere

**Affiliations:** 1 Pediatrics and Child Health, School of Medicine, College of Health Sciences Makerere University, Kampala, Uganda; 2 Department of Surgery, College of Health Sciences Makerere University, Kampala, Uganda; 3 Mulago National Referral Hospital, Kampala, Uganda

**Keywords:** Sickle cell disease, Quality of life, PaedsQL, Pneumococcal vaccine, Caretaker, Sub-Saharan Africa

## Abstract

**Background:**

Sickle cell disease (SCD) is a chronic hematologic disease associated with increased morbidity and mortality. Hemoglobinopathies are the most prevalent genetic disease globally, and SCD is estimated to affect 0.7% of Ugandan. The disease may adversely impact on the quality of life of sickle cell patients. This study aimed to evaluate the health related quality of life (HRoL) of adolescents with SCD.

**Methods:**

This was a mixed-methods study of adolescents with sickle cell disease and their caretakers living in Kampala city, Uganda. All children aged 8–17 years with homozygous sickle cell disease attending the sickle cell clinic at Mulago Hospital during the study period were included in this study. Participants completed the PedsQL™ generic core scales parent-proxy and child self-report questionnaire during a routine clinic visit. HRQoL was the primary outcome measured. Socio-demographics and disease related data were obtained through personal interview with caretakers and reviewing patients’ medical records. Mean scores were used for HRQoL and linear regression for associated factors.

**Results:**

Of the 140 adolescents with SCD included in the study, 40% were male. A total of 95 adolescents (68%) were between the age of 8–12 years with a mean age of 14.25 years. The physical function was assessed slightly higher by adolescents with a mean score of57.5 ± 20.3 compare to caretakers with 52.8 ± 22.1(*p* < 0.001). As assessed by caretakers, physical HRQoL scores were negatively associated with pain about-10.02 CI [− 19.22, − 0.81](*p* = 0.033), whereas it was positively associated with Pneumococcal vaccine with the score of 28.43 CI [16.78,40.09](*p* < 0.001) as assessed by adolescents and 31.37CI [22.22,40.51](p < 0.001) by caretakers. Pneumococcal vaccination impacted positively the psychosocial functioning with a score of 8.67CI [1.51,15.84] (*p* = 0.018) as assessed by children and 15.94 CI [5.50,26.38](*p* = 0.003) as assessed by the caretakers.

**Conclusions:**

This study highlighted that pain was negatively associated with both physical and psychosocial functioning; whereas getting Pneumococcal vaccine was positively associated with both physical and psychosocial functioning as reported by children and caretakers.

## Background

Sickle cell disease (SCD) is a group of inherited red blood cell disorders characterized by the presence of abnormal hemoglobin where there is a substitution of hydrophilic glutamic acid by hydrophobic Valine at position 6 of beta-globin chain. The clinical manifestations are diverse and may include vaso-occlusive, hematological and infectious crisis. Sickle cell disease is a serious global health issue with an estimated 400,000 neonates born with the disease each year and about 300,000 of these with sickle cell anemia [1]. The greatest burden is seen in sub-Sahara Africa where more than 75% of all sickle cell disease occur; this proportion is projected to increase by 2050 [1]. In Uganda, the overall number of children with sickle cell trait is 13.3% and with disease is 0.7% having SCD [1].

Health Related Quality of life (HRQoL) is defined as an extent to which one’s usual or expected physical, emotion and social well-being is affected by a medical condition or its treatment [2]. Previous study found a lower HRQoL to patients with SCD compared to the general adult’s population [3]. In that study, a lower HRQoL was found in several domains by children with SCD and their parents [3, 4].

A number of disease-related factors have been found to affect quality of life (QoL) in children with SCD such as vulnerable socioeconomic status, age of the patient by physical manifestations of SCD notably in the form of abnormal sexual and physical development, type of pathology, family support, beliefs and values [5]. The clinical conditions such as pain, fever, skin redness, swelling, and history of blood transfusion are significant predictors of lower HRQoL in children with sickle cell disease [5]. Swelling has been documented to negatively affect to physical function, vitality, social function, bodily pain, and general health [5].

This study represents the first investigation of SCD patients’ QoL of adolescent population in Uganda. We hypothesized that SCD in adolescents was associated with low HRQoL and demographic and disease factors might have an impact on HRQoL. In this study we aimed to describe the health-related QoL of adolescents with SCD, and examine explanatory predictors that might be associated with QoL.

## Methods

### Study design, population and site

The present study used a mixed-methods study design with quantitative and qualitative methods that included adolescents with SCD and their caretakers, randomly selected from the Outpatient sickle cell clinic at Mulago National Referral Hospital in Kampala, Uganda from February to April 2018.The study sample consisted of children with SCD aged 8–17 years and their caretakers. Inclusion criteria*:* All children aged 8–17 years with homozygous sickle cell disease attending the sickle cell clinic at Mulago Hospital during the study period. Exclusion criteria were: a history of a previous chronic disease (like cancer tuberculosis, HIV); any concomitant serious medical problems (other than those secondary to SCD). A consecutive sampling was done. This process was continued until sample size was reached. Many of our participants were visiting for acute pain and few were in their usual state of health.

### Variables measurement

#### The socio-demographic and disease characteristics

An interviewer administered questionnaire was used to obtain information on the participants and the caregiver’s socio-demographic characteristics (age, gender, date of birth, relationship with child, education, residence and household income) and history of blood transfusion for the last one year. Physical examination findings such as vital signs (temperature, pulse rate, respiratory rate, and oxygen saturation), presence or absence of complications of the disease, such as infection, dehydration and pain, were recorded. The time period reviewed for pain and fever was information within the 30 days. Fever was defined as axillary temperature equal or more than 38 C (101.3 F). Pain: constant cry restlessness, facial expression and verbal report of pain. Anthropometric measurements were taken. Malaria (positive blood slide), hemoglobin electrophoresis, treatment such as Hydroxyurea, Folic acid and Pneumococcal vaccine were obtained by reviewing patients’ medical records.

#### Evaluation of heath quality of life

The acute version of pediatric quality of life questionnaire with 7 days recall was separately administered to parent proxy and children by the research assistant to determine Health Related Quality of Life [6]. The PedsQL™ Generic Core has four domains: physical, emotional, social and school functioning, with child self-report versions for a broad age range (age groups 5–7, 8–12, and 13–18) and parallel parent proxy reports for each age group. The Pediatric quality of life inventory is composed of 23 items comprising 4 dimensions. Higher scores in the inventory indicate better health related quality of life. In this study a 5 point scale was used with scores ranging from 0(never) to 4 (almost always). Likert response scale items are reverse-scored and linearly transformed to a 0–100 scale (0 = 100, 1 = 75, 2 = 50, 3 = 25, 4 = 0), so that higher PedsQL™ scores indicate better HRQoL [7, 8]. The established cut-off scores meaning poor quality of life by using PedsQL™ generic instrument were 69.7 for child self-report and 65.4 for parental proxy report in comparison with general population [9]. The English version was translated by the research assistant.

### Qualitative methods of data collection

Qualitative data were collected using focus group discussions (FGDs) with adolescents and caretakers. Two FGDs discussions were conducted with children and 2 FGDs with their caretakers/parents. For each category one FGD was with those who assessed low quality of life and another one with those who assessed high quality of life for children with SCD. Each FGD had 7–10 participants.

### Statistical analyses

The quantitative data were entered into Epidata version 3.1 and exported to STATA for analysis. For the qualitative component, audio recorded discussions were transcribed and analyzed manually using content thematic approach which involved grouping data according to study themes and sub-themes with guidance from the qualitative researcher on the team. Direct quotations from study participants were used in the presentation of the study findings.

Descriptive statistics such as means and standard deviation (mean ± SD) were used to describe the quantitative variables. Frequencies (N) and percentages (%) were used to describe categorical variables. At univariate and multivariate analysis stage a multiple linear regression model was fit with HRQoL as the dependent variables and only independent variables with *p*-values (p) less than 5% were considered as the significantly associated factors. In multiple linear regression analyses, *p* < 0.05 was considered significant.

### Ethical considerations

Ethical approval was obtained from Makerere University College of Health Sciences, School of Medicine Research and Ethics Committee and the National Council for Science and Technology. Informed consent for data collection and publication was obtained from parents/caretakers, and informed assent from children.

## Results

### Demographic and disease factors

Of the 140 children with SCD included in the study who were all homozygous SS, 40% were male. A total of 95 children (68%) were between the ages of 8–12 years (Table 1), mean age was 14.25 years. Among the caretakers, 53% were above 35 years of age, 91% were female, and 113(84%) had income level of less than 57$ per month. The majority of the children 91/140(65%) came to the hospital with fever and 89/140 (64%) came with pain. Only 35% are on Hydroxyurea, 131(95%) are taking folic acid and 26% have gotten Pneumococcal vaccine. Only 3% got more than 3 blood transfusions in the last one year. Further details of study participants are presented in Table 2.

**Table 1:** Socio-demographic of adolescents with SCD and their caretakers

Adolescents with SCD			Caretakers of adolescents with SCD		
Variables	Frequency(N=140)	Percent	Variables	Frequency(N=140)	Percent
Gender			Gender		
Male	56	40	Male	12	9
Female	84	60	Female	128	91
Age group			Age group		
8-12	95	68	<35	66	47
>=12	45	32	>=35	74	53
Relationship with child			Income level^a^		
Mother	122	87	<=57 $	113	84
Other	18	13	>57 $	22	16
Marital status			Marital status		
Married	-	-	Married	124	89
Single	-	-	Single	16	11

^a^ Some missing variables for income level

**Table 2:** Disease characteristics of adolescents with SCD

Variables Frequency	(N=140)	Percent	Variables	Frequency (N=140)	Percent
Fever			Hydroxyurea^a^		
Yes	91	65	Yes	45	35
Non	49	35	Non	84	65
Malaria^a^			Folic acid^a^		
Yes	13	10	Yes	131	95
Non	123	90	Non	7	5
Pain			Pneumococcal^a^		
Yes	89	64	Yes	36	26
Non	51	36	Non	101	74
Stroke			Blood transfusion		
Yes	20	14	None	117	84
Non	120	86	1-3	20	14
			>3	3	2

^a^Some missing variables for income level, malaria, Hydroxyurea, Folic acid and Pneumococcal vaccine

### The mean HRQoL

As shown in Table 3, the mean score of physical, emotional school and psychosocial functioning were below the recommended cut off as assessed by adolescents. However, social functioning was high as assessed by both adolescents and caretakers but it was not statistically significant. The HRQoL was lower in female in emotional functioning p<0.0005, and social functioning p<0.007 Table 4. Adolescents on pain reported their HRQoL as low in school functioning with p<0.003.The HRQoL was reported high by adolescents taking Hydroxyurea in all domains though it was only significant in physical functioning with a p<0.001. Adolescents having experienced stroke, reported their HRQoL as low but it was not significant in school functioning (*p* = 0.12) and physical functioning (*p* = 0.14) Table 4.

**Table 3:** Mean score of PedsQLTM of adolescents with SCD as assessed by adolescents andcaretakers

Variables	N	Child Means(±SD)	N	Caretaker Means(±SD)	Mean Diff.	p
Total score		62.98(14.5)		63.24(15.9)	−0.26	0.667
Physical functioning	140	57.5 (20.3)	121	52.8 (22.1)	4.7	< 0.001
Emotional functioning	140	61.4 (19.0)	121	67.2 (20.9)	−5.8	0.003
Social functioning	140	74.6 (21.5)	121	73.4 (24.4)	1.2	0.7609
School functioning	140	58.4 (19.9)	121	59.7 (22.5)	−1.3	0.7586
Psychosocial HRQoL	140	64.8 (15.9)	121	67.0 (18.6)	−1.2	0.1216

Psychosocial HRQoL: includes sum of Emotional, Social and School functioning

**Table 4:** Mean score of HRQoL, demographic and disease factors as reported by adolescents

Variables	PhF	p	EmF	p	SocF	P	SchF	p	PsyF	p
Age group(child) Means (±SD)										
> 12	57.8 ± 20.13	0.9018	67.6 ± 16.36	0.0079	78.7 ± 18.73	0.1258	60.6 ± 14.93	0.3065	68.9 ± 16.31	0.0348
≤12	57.3 ± 20.52		58.5 ± 19.58		72.7 ± 22.57		57.4 ± 17.71		62.9 ± 14.37	
Gender (child) Means (±SD)										
Male	57.7 ± 21.85	0.9094	68.1 ± 21.01	0.0005	80.6 ± 17.58	0.0070	58.3 ± 19.05	0.9433	69.0 ± 16.76	0.0105
Female	57.3 ± 19.78		56.9 ± 16.22		70.7 ± 23.05		58.5 ± 15.38		62.0 ± 14.77	
Fever Means (±SD)										
No	54.9 ± 13.19	0.2711	65.9 ± 21.23	0.0385	77.4 ± 2.17	0.2770	56.8 ± 14.17	0.4149	66.7 ± 14.87	0.3070
Yes	58.7 ± 23.23		59.0 ± 17.38		73.1 ± 24.53		59.3 ± 18.19		63.8 ± 16.44	
Pain										
No	61.5 ± 22.3	0.0735	59.21 ± 20.70	0.3073	74.8 ± 22.53	0.9227	65.1 ± 15.18	0.0003	64.8 ± 15.44	0.3771
Yes	55.1 ± 18.83		62.64 ± 18.01		74.5 ± 21.07		54.6 ± 16.69		63.9 ± 16.19	
Malaria Means (±SD)										
No	57.5 ± 21.1	0.2940	59.7 ± 15.53	< 0.001	73.6 ± 20.84	0.0003	56.6 ± 16.8	< 0.001	63.3 ± 14.21	< 0.001
Yes	63.7 ± 8.2		88.5 ± 21.92		95.4 ± 8.77		75.4 ± 8.77		86.4 ± 13.16	
Hydroxyurea Means (±SD)										
No	53.6 ± 18.95	< 0.001	59.5 ± 21.56	0.1179	73.0 ± 24.61	0.2394	56.6 ± 17.48	0.1182	63.1 ± 18.15	0.0866
Yes	69.5 ± 17.86		65.33 ± 15.09		77.6 ± 12.86		61.7 ± 17.29		68.2 ± 11.20	
Folic acid Means (±SD)										
No	71.5 ± 0.17	0.0638	59.3 ± 1.89	0.7564	78.6 ± 9.45	0.6036	62.9 ± 7.56	0.4634	66.9 ± 5.04	0.7127
Yes	56.9 ± 20.45		61.6 ± 19.65		74.2 ± 22.06		58.0 ± 17.28		64.6 ± 16.39	
Pneumococcal vaccine Means(±SD)										
No	56.7 ± 17.84	0.4348	58.3 ± 20.93	0.0011	72.3 ± 23.91	0.0586	59.0 ± 18.96	0.4628	63.2 ± 17.97	0.0608
Yes	59.9 ± 20.54		70.3 ± 8.53		80.3 ± 12.07		56.5 ± 9.47		69.03 ± 7.39	
Stroke Means (±SD)										
No	58.5 ± 21.13	0.1401	63.7 ± 17.05	0.0004	76.1 ± 20.47	0.0441	59.3 ± 16.33	0.1207	66.4 ± 14.86	0.0042
Yes	51.3 ± 13.39		47.8 ± 24.52		65.7 ± 25.83		52.0 ± 19.43		55.5 ± 19.04	

*PhF* Physical functioning, *EmF* Emotional functioning, *SocF* Social functioning, *SchF* School functioning, *PsyF* Psychosocial functioning

### Disease factors and HRQoL

We found that a change of one unit from not receiving Pneumococcal vaccine to receiving it increases the physical functioning by 28 and 31 points (*p* < 0.001) in both adolescents and caretakers respectively. Having gotten more than 3 blood transfusions impacted positively on the physical functioning as assessed by adolescents and caretakers (*p* = 0.002) at multivariate analysis (Table 5).

**Table 5:** Disease factors and physical HRQoL of adolescents with SCD at multivariate regression analysis (adolescents and caretakers report)

Variables	Physical HRQoL			
	Adolescents’ response Estimate (95% CI)	P	Caretakers’ response Estimate (95% CI)	p
Malaria				
No	1.00		100	
Yes	32.39(20.14,44.63)	< 0.001	37.05(27.95,4.14)	< 0.001
Hydroxyurea				
No	1.00		1.00	
Yes	−0.84(−10.98,9.29)	0.869	5.30(−5.59,16.20)	0.336
Folic acid				
No	1.00		1.00	
Yes	−28.76(− 45.41,-12.10)	0.001	− 46.17(− 65.96,-26.39)	< 0.001
Pneumococcal vaccine				
No	1.00		1.00	
Yes	28.43(16.78,40.09)	< 0.001	31.37(22.22,40.51)	< 0.001
Pain				
No	1.00		1.00	
Yes	−4.70(− 14.35,4.95)	0.336	− 10.02(− 19.22,-0.81)	0.033
Fever				
No	1.00		1.00	
Yes	24.39(15.03,33.74)	< 0.001	30.67(23.5,37.84)	< 0.001
Stroke				
No	1.00		1.00	
Yes	9.15(−0.75,19.05)	0.070	−29.34(−40.69,18.59)	< 0.001
Blood transfusion				
None	1.00		1.00	
1–3	6.63(−6.60,19.86)	0.323	3.48(−8.40,15.36)	0.562
> 3	31.17(11.6.2,50.72)	0.002	24.03(8.83,39.23)	0.002

It was found that Hydroxyurea, pain, stroke, and more than 3 blood transfusions impacted negatively on the psychosocial functioning as assessed by both adolescents and their caretakers. However, Pneumococcal vaccination and history of 1–3 blood transfusions had a positive impact on psychosocial functioning (Table 6).

**Table 6:** Disease factors and psychosocial HRQoL of adolescents with SCD at multivariate regression analysis (adolescents and caretakers report)

Variables	Psychosocial HRQoL			
	adolescents’ response Estimate (95% CI)	p	Caretakers’ response Estimate (95% CI)	p
Malaria				
Yes	38.74(31.21,46.30)	< 0.001	17.39(7.00,27.77)	< 0.001
Hydroxyurea				
Yes	−12.57(−18.80,-6.34)	< 0.001	−29.165(−41.35,-16.98)	< 0.001
Folic acid				
Yes	1.27(−8.97,11.51)	0.806	−11.62(−33.63,10.38)	0.296
Pneumococcal vaccine				
Yes	8.67(1.51,15.84)	0.018	15.94(5.50,26.38)	0.003
Pain				
Yes	−4.37(−10.30,1.56)	0.147	−29.17(−39.39,-18.94)	< 0.001
Fever				
Yes	16.06(10.30,21.01)	< 0.001	4.53(−3.48,12.56)	0.264
Stroke				
Yes	−6.78(− 12.87,-0.70)	0.029	−29.34(−40.69,18.59)	< 0.001
Blood transfusion				
1–3	13.89(5.76,22.03)	0.001	−26.46(−40.00,-12.91)	< 0.001
> 3	−28.28(−40.30,-16.26)	< 0.001	−62.63(−79.52,-45.74)	< 0.001

### Qualitative result

With regard to qualitative results, all adolescents and their caretakers in group discussions mentioned missing school due to frequent pain and hospital visits which translated into poor school performance as key factors negatively affecting the HRQoL for adolescents with SCD. Many adolescents talked about how their academic performance had declined due to SCD as they explained:

“When you look at the academic side my performance is affected because I get attacks during examinations and the performance is poor because the society does not look into us as a special case to give special examinations” (FGD adolescents with low HRQoL).

“Pain and coming to the hospital for care stops me from going to school…” (FGD adolescents with low HRQoL).

Most of the adolescents and their caretakers reported stigma to be a major factor affecting HRQoL for adolescents with SCD. Some adolescents felt they had been stigmatized on the basis of how they looked due to SCD and were often excluded from playing with other children as one of them explained: “For me children at school abuse me. They say that I have eyes like for a wild cat because they are yellow. They tell me that they can’t play with me because they don’t want to associate with sicklers. This makes me feel bad and I cry” (FGD with adolescents with low HRQoL).

Another adolescent added: “Even me they don’t like me at school. They say that I should take away my sickle cell yet when they want to learn something they come and ask me but for me I tell them the answers but even in class I perform better than them” (FGD caretakers of adolescents with low HRQoL).

Most adolescents reported that SCD limits them from taking part in community and household activities as well as play which negatively affected their quality of life.

“For me I can’t participate in sports yet I like football, I do not participate in sports and games because I cannot compete with other children so we lose our skill and yet we always love our hobbies” (FGD adolescents with low HRQoL).

“For me I can’t do heavy work because I am weak. Sometimes I faint and my mother doesn’t allow me to carry heavy things. When I do heavy work I start feeling pain and sometimes they end up bringing me to the hospital” (FGD adolescents with low HRQoL).

## Discussion

SCD has been widely researched on in sub-Saharan Africa, but there is a paucity of studies on the impact of the disease on the school, emotional, physical, and social well-being of the patients despite the high prevalence of SCD in sub-Saharan Africa. In this study, we found that the HRQoL was low in physical functioning, emotional functioning and school functioning as assessed by adolescents, and was also low in physical functioning and school functioning as assessed by the caretakers.

The physical and emotional functioning are lower than the study done by Joanita et al. [10]; this could be explained by the fact that their study was conducted in a higher income country, where by children with SDC are likely to have better care. This finding can also be explained by cultural differences where level of social support, coping style and perception of illness are different as compared to the developed countries.

Qualitative results also indicated that SCD affects negatively school functioning. In addition most of the study participants in FGDs reported stigma to be a factor affecting their emotional HRQoL.. Most adolescents reported having a problems with their physical functioning such as in the form of being unable to play and do home activities due to SCD and related complications.

Our findings on low HRQoL among children with SCD are consistent with those of previous studies [11–13]. The study done by Mohammad and Kamfar regarding the association between disease severity, depression, social support, and the combined and/or singular effect on HRQoL in children with SCD, found a significant impairment of QOL in all domains among children with SCD [14].

Panepinto et al. used the PedsQL™ 4.0 and concluded an impaired HRQoL in children with SCD, even after considering the potential effect of family income on HRQoL. In another study, Dale et al. found an overall HRQoL lower in children with SCD and their parent than in healthy children and their parents, a finding that matched our results [15].

Gender influences HRQoL of adolescents with SCD. A previous study using the Child Health Questionnaire found that children /reported statistically significant gender differences in HRQoL with diminished scores on most functioning scales for female subjects [16]. Gender differences in pain have been described; the frequency of pain is higher among female [17].

Adolescents with SCD who are taking Hydroxyurea therapy report better physical functioning than those not taking the medication. This is consistent with the study done by Coutney D. et al were they found a better physical functioning in children taking Hydroxyurea [18].The study done by S. M. Badawy et al, showed that patients on Hydroxyurea with no recall barriers had better HRQoL [19]. This is because of the impact of a disease modifying therapy (Hydroxyurea) on the HRQL of children with SCD. It is well known that Hydroxyurea therapy decreases the frequency of vaso-occlusive episodes.

Stroke influence the HRQoL of adolescents with sickle cell disease. The study done by Chia Yeh Chou also found that, the psychosocial and physical factors impacted stroke-specific HRQoL [20]. The characteristics of individual patients, their social environment, the specific type of care provided and the experience of disability are factors that influence the impact of stroke on the HRQoL level. The predictors of poor QoL post-stroke include: depression, medical comorbidities, functional status and perceived social support [21].

Adolescents in pain rated their HRQoL as low in school functioning. Anie found that pain experience accounted for12.3% of hospital and general practice service use, independently of age, sex, number of SCD complications and hemoglobin levels [22]. We found also that pain impacted negatively on the physical and psychosocial HRQoL. This is in consistence with the study done by Carlton et al. where they found that pain negatively influenced child reports on almost all functioning [23]. In our study children narrated how pain limited them from attending school as well as participating in play activities which negatively affected their quality of life. Ludwig et al. found that executive functioning is an important factor in understanding the relationship between pain coping and HRQoL in youth with SCD [24].

A study done in US by S. K.Ballas et al. found pain as a predictor of quality of life QoL outcomes, in physical and social functioning [25]. Another study found an impaired QoL in patients with acute and chronic pain more than any other disease-related complication [26].

At multivariate analysis in this study, more than 3 blood transfusions were associated with increase in physical functioning of adolescents with SCD (Table 5). Reducing anemia by giving blood transfusion, painful crisis will reduce, thus improving the HRQoL. This finding is in agreement with the findings of a randomized trial study done by Laurent et al. where blood transfusion was found to improve health among children with SCD. The parents reported that their transfused children had better physical health functioning a year later [27]. With regard to psychosocial functioning, the blood transfusion was assessed by adolescents and their caretakers to have a negative influence on adolescent’s quality of life. This might be due to the painful procedures that traumatize children when getting blood transfusion, though it physically helps them.

It was found in this study that Hydroxyurea influenced positively the physical functioning as reported by caretakers; but it was not statistically significant. Treatment of SCD patients with Hydroxyurea improves their clinical and hematological characteristics. Thornburg et al. in Carolina also found that Children with SCD who were taking Hydroxyurea therapy reported better physical functioning and overall HRQoL than those not taking the medication despite disease severity and older age [28]. Tshilolo et al. found that Hydroxyurea used reduced the incidence of vaso-occlusive events, infections, malaria, transfusion and death [29]. In the study done by McClish et al. data showed that Hydroxyurea can also improve some measures of QoL including general health perception and pain recall. The effect was especially evident in patients with sustained HbF (fetal hemoglobin) response to Hydroxyurea.

With regard to psychosocial functioning, we found a negative impact of Hydroxyurea on psychosocial domain with a *p* < 0.001 as assessed by adolescents and caretakers. The study done by Thornburg et al. in Carolina found no differences between groups of patients on Hydroxyurea and those not on Hydroxyurea in the psychosocial summary score or its individual domains [28]. The lack of demonstrable effect of Hydroxyurea on certain QoL measures may be the result of issues inherent in patients with moderate to severe disease who were already debilitated and had irreversible effects of their disease or poor adherence to drug; Sherif et al. found that female had worse QoL score and were poorly adhered to Hydroxyurea [30]. In the study done by Badawy et al. it was found that beliefs about Hydroxyurea correlated with HRQoL score and adherence [31]. In the qualitative part of the study, children reported difficulties in adhering to daily drugs which negatively affected their quality of life. It is important to consider also the effect of Hydroxyurea in increasing HbF as found by the study done by McClish et al. [32].

Pneumococcal vaccination was associated with increase in physical functioning and psychosocial functioning as assessed by both adolescents and their caretakers (Tables 5 and 6). Pneumococcal vaccine reduces the frequency of bacterial infections thus improving the HRQoL for children with SCD [33].

In a study done by Gayatri et al. concerning initial outcomes of a comprehensive care model for sickle cell disease among a tribal population in rural western India there was a reduction in the proportion of patients with ≥3 pain crises per year (35.4 to 9.8%, *p* < 0.001) and the proportion of patients with ≥1 hospitalization per year (56.7 go 36.0%, p < 0.001) in patients who got Pneumococcal vaccine and Hydroxyurea [34].

### Limitation of the study

In this study, we enrolled patients who were only in the Sickle clinics of the Mulago National Referral Hospital, Kampala, Uganda, which may have resulted in a bias towards enrolling patients who are severely sick. We extracted data on some variables from clinic records some of which had missing values. We did a consecutive sampling. This process was continued until sample size was reached; many of our participants were visiting for acute pain and few were in their usual state of health. That might have impacted HRQoL. We also had lack of qualitative validation of the translated version.

## Conclusion

This study was done to assess the HRQoL of adolescents with SCD as assessed by adolescents and their caretakers. We found that adolescents with SCD have low HRQoL as assessed by participants. The mean physical, emotional, school and psychosocial functioning were below the cut-off as assessed by adolescents with SCD.

We found also that Pneumococcal vaccination and getting more than 3 blood transfusions were positively associated with physical functioning. Taking Hydroxyurea, having stroke and getting more than 3 blood transfusions negatively influenced the psychosocial functioning of adolescents with SCD. Adolescents with SCD experienced regular pain, problems with growth, stigma and missed school which negatively affected their HRQoL. We recommend further studies with long term prospective to identifying causal association of above reported factors.

## Abbreviations

$: United States dollar.
FGD: Focus group discussions
HbF: Fetal hemoglobin
HRQoL: Health-related quality of life
PedsQL™: Pediatric Quality of Life Inventory
QoL: Quality of life
SCD: Sickle cell disease
